# Editorial Notes

**Published:** 1882-02

**Authors:** 


### EDITORIAL NOTES.

We regret to announce the serious illness of Dr. Wm. Gould, of this city, which has confined him to his bed for several weeks.

An important change has taken place in the firm of Geo. I. Thurstone & Co., druggists, 416 Main street, Buffalo, Mr. C. M. Lyman retiring, and his place filled by E. H. Hutchinson. We wish the new firm deserved success.

Under Clinical Reports in our last issue we inadvertently made the prescription recommended by Dr. Boardman for herpes zoster read two drams of carbolic acid to two ounces of olive oil, when it should have read, two scruples of carbolic acid. This correction should be heeded by the profession in using this valueable prescription.

The present term of lectures at the Medical College is drawing to a close, the commencement being announced to take place probably on Tuesday, Feb. 21. The present senior class is made up of excellent material, and will prove a valuable accession to the Alumni of the college and to the profession into which they are soon to be introduced.

We are pained to announce the death of Mrs. Dr. White, which occurred January 23, not four months after the death of her distinguished husband. The Alumni of the college and the profession will join in the expression of deep sorrow that this estimable lady, borne down under her severe affliction, and also under severe physical suffering, should be so soon taken away.

In our last number, we referred to the probable retirement of Dr. A. H. Briggs as Health Officer. We are gratified to announce that the Board of Health have made a wise selection in appointing to fill this important position Dr. W. C. Phelps, who brings a large experience and good executive ability to the discharge of its duties. We express the sentiment of the profession when we say that a better appointment could not have been made.

The epidemic of variola, prevailing in this city, seems to be effectually checked, thanks to the energy of the Health officers, to whose efforts great credit is due. We chronicle with pleasure the eminent success of our friend, Dr. Marcley, to whom the care of all cases at North Buffalo has been intrusted, in the treatment of this loathsome disease. It only remains for the Board of Health to amply compensate him for his labors.

The Board of Censors desire to call the attention of the profession to the fact that they are now ready to test the new medical law. They therefore seek information concerning any instance of persons practicing illegally with evidence to prove the same. The apathy of the profession in this very important matter may well be the subject of severe criticism. We hope hereafter the efforts of the Board of Censors to suppress quackery will be earnestly seconded by the profession generally,

The secretaries of the International Medical Congress, which met in London last August are to be congratulated upon the promptness with which the transactions are to appear. These are contained in four royal octavo volumes, of 2,400 pages, and relating as they do to almost every branch of medicine and surgery, form indeed quite an encyclopædia. Each member is, of course, entitled to a set, and those who are not, can obtain the volumes by purchase. Moreover, as few physicians would care for all the papers, but many would probably wish to read those of special interest to themselves, the very wise plan has been adopted of publishing in compact form a limited number of the transactions of each section separately. These may be obtained from a Mr. Kolckmann, 2 Langham Place, London, at a moderate price—six or seven shillings, and will probably be much in demand.

